# Multidisciplinary approach to cancer care in Rwanda: the role of tumour board meetings

**DOI:** 10.3332/ecancer.2022.1515

**Published:** 2023-03-06

**Authors:** Brandon A Niyibizi, Eulade Muhizi, Daniella Rangira, Diane A Ndoli, Innocent N Nzeyimana, Jackson Muvunyi, Magnifique Irakoze, Madeleine Kazindu, Alex Rugamba, Khadidja Uwimana, Yuanzhen Cao, Eulade Rugengamanzi, Jean de Dieu Kwizera, Achille VC Manirakiza, Fidel Rubagumya

**Affiliations:** 1 Rwanda Cancer Relief, Kigali, Rwanda; 2 Department of Oncology, Rwanda Military Hospital, Kigali, Rwanda; 3 School of Medicine, College of Medical and Health Sciences, University of Rwanda, Kigali, Rwanda; 4 Department of Oncology, Muhimbili University of Health and Allied Sciences, Dar es Salaam, Tanzania; 5 Department of Urology, Butare University Teaching Hospital, Huye, Rwanda; 6 Department of Internal Medicine, Dartmouth–Hitchcock Medical Center, Lebanon, NH 03766, USA; 7 Department of Gynecology, Kigali University Teaching Hospital, Kigali, Rwanda; 8 Unit of Oncology, Department of Internal Medicine, King Faisal Hospital, Kigali, Rwanda; 9 Department of Internal Medicine, Ruhango Provincial Hospital, Ruhango, Rwanda; 10 Department of Gynecology, Munini District Hospital, Nyabihu, Rwanda; 11 Department of Internal Medicine, Gisenyi Hospital, Rubavu, Gisenyi, Rwanda; 12 Ocean Road Cancer Institute, Dar es Salaam, Tanzania; 13 Department of Epidemiology, Dalla Lana School of Public Health, University of Toronto, Toronto M5T 3M7, Canada

**Keywords:** cancer, multidisciplinary team, tumour board meeting, low and middle income countries, Rwanda

## Abstract

**Introduction:**

Cancer treatment is complex and necessitates a multidisciplinary approach. Tumour Board Meetings (TBMs) provide a multidisciplinary platform for health care providers to communicate about treatment plans for patients. TBMs improve patient care, treatment outcomes and, ultimately, patient satisfaction by facilitating information exchange and regular communication among all parties involved in a patient’s treatment. This study describes the current status of case conference meetings in Rwanda including their structure, process and outcomes.

**Methods:**

The study included four hospitals providing cancer care in Rwanda. Data gathered included patients’ diagnosis, number of attendance and pre-TBM treatment plan, as well as changes made during TBMs, including diagnostic and management plan changes.

**Results:**

From 128 meetings that took place at the time of the study, Rwanda Military Hospital hosted 45 (35%) meetings, King Faisal Hospital had 32 (25%), Butare University Teaching Hospital (CHUB) had 32 (25%) and Kigali University Teaching Hospital (CHUK) had 19 (15%). In all hospitals, General Surgery 69 (29%) was the leading speciality in presenting cases. The top three most presented disease site were head and neck 58 (24%), gastrointestinal 28 (16%) and cervix 28 (12%). Most (85% (202/239)) presented cases sought inputs from TBMs on management plan. On average, two oncologists, two general surgeons, one pathologist and one radiologist attended each meeting.

**Conclusion:**

TBMs in Rwanda are increasingly getting recognised by clinicians. To influence the quality of cancer care provided to Rwandans, it is crucial to build on this enthusiasm and enhance TBMs conduct and efficiency.

## Background

Cancer care is complex and requires a multidisciplinary approach [1]. Tumour Board Meetings (TBMs) or case conferences provide a multidisciplinary platform to facilitate communication between health care providers about treatment planning for patients [1]. These meetings also serve a valuable educational role for both consultants and trainees. Ideally, a TBM includes specialists from medical oncology, radiation oncology, surgical oncology, pathology, diagnostic imaging, nursing, nutrition and social work; this largely varies depending on the institution and available workforce [2]. TBMs can be disease site-specific or general in nature.

TBMs improve patient care, treatment outcomes and ultimately patient satisfaction by facilitating information exchange and regular communication between all those involved in a patient’s treatment. The meetings allow for a consensus plan to be reached among all participating specialists. Evidence has shown that TBMs improve patient outcomes for cancers of colon, oesophagus, breast and lung [3–6]. In this regard, they function as a review of care quality and form the basis for quality improvement. Additionally, TBMs have been shown to improve adherence to clinical practice guidelines [7] and lead to modification of existing treatment protocols and prompt creation of new ones [8]. TBMs also provide an opportunity for education and learning for participants and trainees as complex issues in patient care are discussed and the rationale behind treatment decisions is explained [9, 10].

TBMs are well established globally [1], especially in high-income countries like the United Kingdom [6, 11, 12], North America [13–15], Europe [16] and Asia [17]. TBMs may be particularly helpful in low- and middle-income countries (LMICs), where patients often present with more advanced cancer requiring multi-modality treatment. In Mozambique, TBMs were found to be cost-effective as evidenced by cost per quality‐adjusted life year when two cohorts of breast cancer patients were compared as pre- and post-TBM implementation [18]. Several other sub-Saharan African (SSA) countries have implemented TBMs both in person and, recently, online [19]. While most TBMs in SSA are general in nature, there is a trend towards site-specific and speciality-specific TBMs [19, 20]. However, some cancer centres in LMICs lack the necessary resources to organise TBMs; this is especially true in sub-Saharan Africa where there is a lack of multidisciplinary support from oncology specialists, oncology surgeons, pathologists and other key personnel as well as a lack of protected time for clinicians to attend TBMs [21–23].

The first cancer centre in Rwanda was established in 2012 [24], and due to a lack of formally trained oncologists, the centre relied on TBMs with colleagues in the United States [25]. This tradition, however, did not spread to later-established cancer centres, particularly those affiliated with university teaching hospitals [26]. To better understand how TBMs can be implemented in the African context, we launched and implemented a quality improvement feasibility study of TBMs in four University Teaching Hospitals in Rwanda. This study describes the current status of case conference meetings in Rwanda including their structure, process and outcomes.

## Methodology

### Study design and settings

Data were collected prospectively from March 2021 to June 2022. The study included four referral hospitals: King Faisal Hospital (KFH), Rwanda Military Hospital (RMH), Kigali University Teaching Hospital (CHUK) and Butare University Teaching Hospital (CHUB). These hospitals are among the five hospitals in Rwanda providing cancer care. These facilities offer pathology, radiology and surgical oncology services. Among the included facilities, only KFH and RMH offer chemotherapy services, and only RMH offers radiotherapy services.

### Tumour board meetings

Each hospital conducted weekly TBMs on their most convenient day. Except for a few oncologists who attended TBMs at other hospitals, all participants were hospital employees and rotating trainees. Generally, participants included oncologists, radiologists, pathologists, surgeons (i.e., General, ENT, urologists, neurosurgeons, gynaecologists etc.), residents and medical students. In all TBMs, the oncologist led the discussion either in person (RMH and KFH) or virtually (CHUB and CHUK) due to a lack of an on-site oncologist. Before TBMs took place, de-identified cases to be discussed were shared with all participants for review via emails or WhatsApp group. It was up to the presenting department and clinicians to decide which cases would be presented, but the majority of the cases were a combination of newly diagnosed and difficult cases. Before the meeting, pathology slides and diagnostic images were reviewed with the concerned clinicians by the radiologist and the pathologist, respectively, and then they were projected and reviewed once more during the TBMs with inputs from all participants. Most times, residents in different fields presented the cases, and attendings/consultants gave their views on the case. The secretary of the meeting wrote down all recommendations in pre-designed TBM registry. Due to the lack of resident oncologists at two sites (CHUK and CHUB), TBMs were conducted virtually so that oncologists could participate.

### Data collection

This study was approved by the Rwanda National Ethics Committee and respective Institutional Review Boards. A research assistant at each site attended these meetings and collected data. Data gathered included patients’ diagnosis, number of attendance and pre-TBM treatment plan, as well as changes made during TBMs, including diagnostic and management plan changes. For confidentiality purposes, each case was coded by a unique identifier. Change in diagnosis included change in tumour type and/or significant findings during pathology and radiological images review or recommendation for further workups. Changes in treatment included treatment modality revisions (e.g. change from chemotherapy to radiation or from surgery to chemotherapy or no therapy). Additionally, records were kept of whether a case was brought to TBM for total management input or input on a component of the treatment plan.

### Statistical methods

Data from secured Google Forms were imported into Microsoft Excel and then to IBM SPSS, version 27.0 for Windows (IBM Corp) for statistical analysis. Descriptive statistics were reported. For continuous variables, mean or median and interquartile ranges were reported. For categorical variables, frequencies and percentages were calculated.

## Results

### Total meetings and cases presented

**Figure 1.**
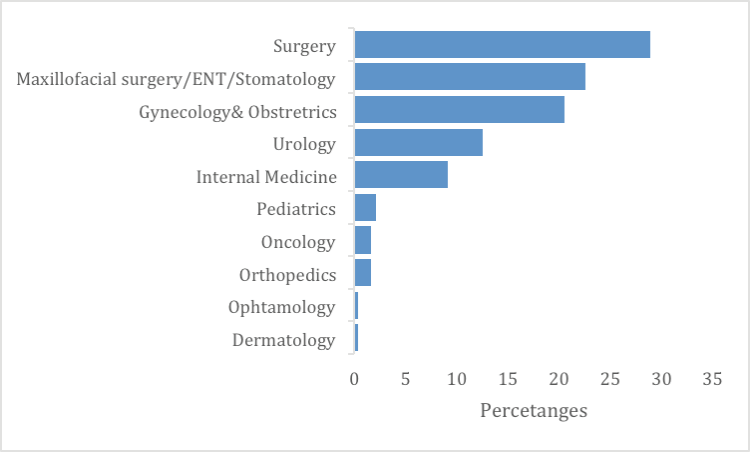
TBM case presentation per departments/speciality.

**Figure 2.**
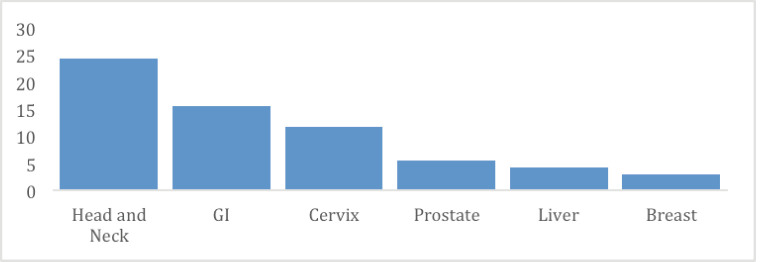
Cases presented at TBM per disease site.

From March 2021 to June 2022, a total of 128 meetings took place in the four hospitals. RMH conducted 45 (35%) meetings, KFH had 32 (25%), CHUB had 32 (25%) and CHUK had 19 (15%). Two hundred and thirty-nine cases were presented: 101 (42%) from RMH, 56 (23%) from KFH, 44 (18%) from CHUK and 38 (16%) from CHUB (Table 1). In all hospitals, General Surgery 69 (29%) presented the highest number of cases, followed by Maxillofacial/ENT/Stomatology 54 (23%) and Gynaecology and Obstetrics 49 (21%) (Figure 1). The most presented disease site was head and neck 58 (24%), followed by gastrointestinal 28 (16%), cervix 28 (12%), prostate 13 (5%); liver 10 (4%) and breast 6 (3%) (Figure 2).

### Reasons for TBM presentation

**Figure 3.**
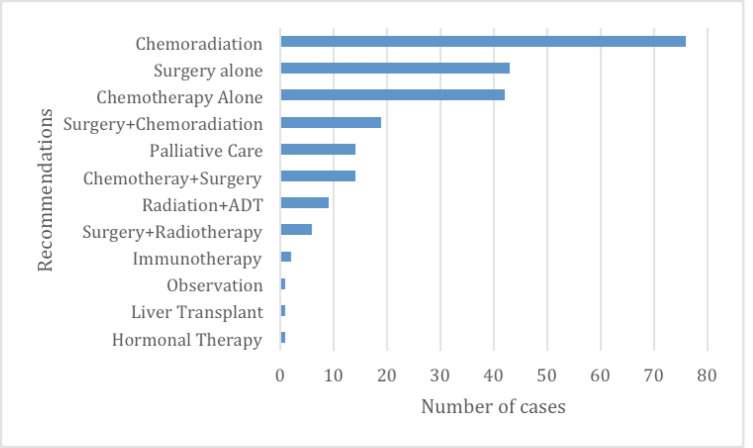
Management recommendations post-TBMs.

Overall, 85% (202/239) of the presented cases sought inputs from TBM on the management plan (i.e. surgery, chemotherapy and radiation); 21% (49/239) sought input on diagnosis and staging (pathology and radiology) and 1% (3/239) was for education purposes. Among those cases presented for management input, 40% (81/202) did not have a pre-TBM management plan and relied entirely on TBM for the treatment plan. Among those with pre-TBM management plans, 21% (30/202) had treatment plans changed during TBMs. Overall TBMs resulted in a variety of management recommendations for cases that were adequately captured, including surgery alone (43/239), chemotherapy alone (42/239), chemoradiotherapy (76/239), surgery and chemoradiation (19/239), chemotherapy and surgery (14/239), observation (1/239), liver transplant (1/239), immunotherapy (2/239), hormonal therapy (1/239), palliative care (14/239) and surgery and radiotherapy (6/239) (Figure 3). For those presented for diagnostic input, 10% (5/49) cases had diagnostic changes (i.e. stage of the disease, pathology grading, other pathology-related prognostic features) and 6% (3/49) had histology diagnosis changes (i.e. histologic type) (Table 2).

**Figure 4.**
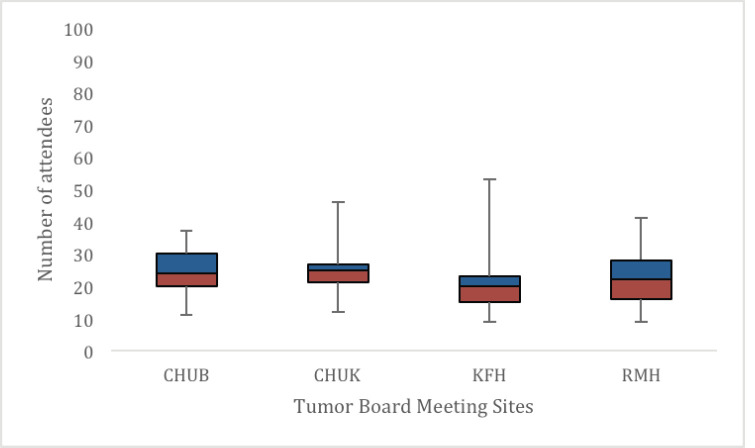
Weekly attendance per TBM site. The horizontal line represents the median score, the coloured boxes represent the interquartile range and the vertical line represents the range.

*Tumour board leadership and attendance* The median attendance varied per TBM site per week with CHUB being 24 (IQR 10), CHUK 25 (IQR 5.5), KFH 20 (IQR 8) and RMH 22 (IQR 13) (Figure 4). On average, at least two oncologists, two general surgeons, one pathologist and one radiologist attended each meeting (Table 3).

The TBM also served as an educational platform for physicians in training with an average of five residents and four medical students attending each meeting. Although most cases were presented by surgeons of various specialities, all clinicians (i.e. physicians, surgeons and nurses) and allied staff (e.g. radiotherapy technician) participated in the discussion and in coming up with final treatment plans.

## Discussion

Our study aimed at evaluating the status of TBMs in Rwanda, including key structure and processes. To the authors’ knowledge, this is the first study to report on the status of multidisciplinary tumour boards in Rwanda. Several important findings have emerged. First, most cases presented at TBM sought input on management plans. Second, while some cases had pre-TBMs plan, more than a quarter of cases solely relied on TBMs for management plan. Third, although several specialities presented cases during TBM, the pre-dominant group of presenters were surgeons. Finally, TBMs were attended by diverse specialities and trainees.

Our findings demonstrate a strong belief and understanding of the importance of multidisciplinary approach to cancer care by cancer treating clinicians in Rwanda. Despite several existing challenges in cancer care such as shortage of personnel, lack of time, the attitude of consulting colleagues will improve the quality of cancer care [27]. The dominance of surgeons (i.e. ENT, Gynaecology, urology, neurosurgery and maxillofacial) in case presentation is a good sign as they are usually the first contact for patients with cancer especially at tertiary hospitals where they are referred for biopsy and primary surgery [28, 29]. While we did not collect this data, most surgeons brought cases to TBMs before surgery for discussion to decide for neoadjuvant treatment versus upfront surgery and others were brought for adjuvant treatment decisions. In addition, our findings demonstrate a multidisciplinary approach, as evidenced by the diverse backgrounds of specialists who attended TBMs in the four sites. Diverse specialities attending TBMs enrich discussion, thereby enhancing the quality of care provided to cancer patients [30].

While this study’s aim was to understand the structure and process of TBM in Rwanda, studies in similar low-resource settings have looked at the impact of TBMs and showed that establishment of TBM was cost-effective and might improve survival among patients who are discussed in these meetings [18]. A study in Botswana studied the impact of TBM among patients with cervical cancer and found reduction in time from biopsy to start of radiotherapy for cases that were discussed in TBMs [31]. These findings support the establishment and scaling up of TBM in Rwanda and other low-resources settings. While TBM benefits all types of cancers, they are known to be more beneficial for rare clinical scenarios and complex cases [32]. In high-income countries, TBM is a requirement for management of cancer patients and accreditation and regarded as Continuous Medical Education (CME) for physicians and students [33, 34].

Contrary to our findings which shows 37% diagnosis and treatment plan changes post-TBMs, other institutional studies have reported changes in diagnosis and treatment plans for about 50% cases presented at TBM [35–38]. This may be due to the country’s lack of some diagnostics and treatment options such as interventional radiology, nuclear medicine (such as PET scans), targeted therapy and radiosurgery, among others. Our study did not investigate whether recommendations suggested during TBM were enacted but studies elsewhere show that about 84% of TBM recommendations are followed, which shows that TBMs impact patient care [38]. The cornerstone of a functioning tumour board is the multidisciplinary nature and ability for all attendees to voice their opinion. The lack of some disciplines and specialities in most hospitals in LMICs makes it difficult to convene all needed specialities physically in one room. However, with technology advancements, clinicians have the option to participate virtually. Virtual tumour boards are a great alternative to physical meetings and have shown to improve the efficiency of multidisciplinary tumour boards [38, 39]. Two of four participating sites in our study implemented a virtual TBM to improve attendance and connect to cancer specialists who could not attend these meetings physically. In several settings, TBM is considered as an education activity for medical students, residents and fellows and offers CME for specialists [40]. Our study findings show a variety of attendees including medical students and we view this as a positive trend towards better oncology education in Rwanda. The primary limitation of our study is that we did not ascertain whether changes suggested in the TBM were implemented and document reasons for non-implementation. Another potential limitation is that the study took place during the COVID-19 pandemic and it is unclear whether TBM participation and cases in the post-pandemic setting would be appreciably different.

This feasibility study has demonstrated the importance of multidisciplinary collaborations, the need for accurate and up-to-date patient information, the requirement for regular meetings to track patients’ progress after TBMs and the documentation of whether TBM decisions are implemented. Moreover, this exercise highlighted the need for administrative personnel to personally document all TBM decisions. As a direction for the future, it is essential to incorporate emerging technologies to improve the tumour board experience. Additionally, it is essential that the tumour board process emphasises patient participation and shared decision-making.

## Conclusion

The complexity of cancer care leads to the requirement of multidisciplinary approaches for the provision of more effective and high-quality care. However, as more TBMs are established in low-resource settings, it is important that their structure, conduct, barriers and facilitators be documented. Challenges to implementation of TBM in low-resource settings include the lack of some specialities, limited resources and infrastructure to implement TBM suggestions and limited time due to other competing responsibilities; these issues should be considered before establishing any TBM. Cancer centres in Rwanda and other SSA countries should support each other and create virtual TBM especially where no adequate specialities needed for a successful TBM.

## Authors’ contributions

Conception and design: Fidel Rubagumya, Brandon A Niyibizi.

Administrative support: Innocent N Nzeyimana, Magnifique Irakoze, Jackson Muvunyi, Achille VC. Manirakiza, Brandon A Niyibizi.

Collection and assembly of data: Madeleine Kazindu, Jean De Dieu Kwizera, Alex Rugamba, Eulade Rugengamanzi, Eulade Muhizi.

Data Analysis and interpretation: Eulade Muhizi, Eulade Rugengamanzi, Brandon A Niyibizi.

Manuscript writing: Fidel Rubagumya, Brandon A Niyibizi, Daniella Rangira, Achille Manirakiza, Diane A Ndoli.

Final approval: All.

## Conflicts of interest

The authors declared no conflicts of interest.

## Support

The TBM project was supported by the International Cancer Institute (ICI), Eldoret, Kenya.

## Figures and Tables

**Table 1.** Tumour board meetings and cases presented at each hospital.

Hospitals	Number of meetings	Cases presented
	***N* (%)**	***N* (%)**
RMH	45 (35)	101 (42)
KFH	32 (25)	56 (23)
CHUB	32 (25)	38 (16)
CHUK	19 (15)	44 (18)
Total	128 (100)	239 (100)

**Table 2.** Reason for case presentation at TBM and TBM changes.

Reason for case presentation	Number of cases *N* (%)	Number of cases with TBM changes *N* (%)
Seeking input on therapeutic plan	202 (85)	30 (21)
Seeking input on diagnosis	49 (21)	8 (16)
Educational purpose	3 (1)	-

**Table 3.** Average attendance per speciality per meeting.

Speciality	Average number of attendants per meeting
Oncologist	2
General surgeon	2
Pathologist	1
Radiologist	1
Resident	5
Medical students	4
Internist	1
Gynaecologist	1
Neurosurgery	1
Urologist	1
Oncology nurse	1
ENT surgeon	1

## References

[1] El Saghir NS, Charara RN, Kreidieh FY (2015). Global practice and efficiency of multidisciplinary tumor boards: results of an American society of clinical oncology international survey. J Glob Oncol.

[2] Wright FC, De Vito C, Langer B (2007). Multidisciplinary cancer conferences: a systematic review and development of practice standards. Eur J Cancer.

[3] Stephens MR, Lewis WG, Brewster AE (2006). Multidisciplinary team management is associated with improved outcomes after surgery for esophageal cancer. Dis Esophagus.

[4] Coory M, Gkolia P, Yang IA (2008). Systematic review of multidisciplinary teams in the management of lung cancer. Lung Cancer Amst Neth.

[5] Kesson EM, Allardice GM, George WD (2012). Effects of multidisciplinary team working on breast cancer survival: retrospective, comparative, interventional cohort study of 13 722 women. BMJ.

[6] Taylor C, Munro AJ, Glynne-Jones R (2010). Multidisciplinary team working in cancer: what is the evidence?. BMJ.

[7] Abraham NS, Gossey JT, Davila JA (2006). Receipt of recommended therapy by patients with advanced colorectal cancer. Am J Gastroenterol.

[8] Lutterbach J, Pagenstecher A, Spreer J (2005). The brain tumor board: lessons to be learned from an interdisciplinary conference. Oncol Res Treat.

[9] Foster TJ, Bouchard-Fortier A, Olivotto IA (2016). Effect of multidisciplinary case conferences on physician decision making: breast diagnostic rounds. Cureus.

[10] Gross GE (1987). The role of the tumor board in a community hospital. CA Cancer J Clin.

[11] Lamb BW, Jalil RT, Shah S (2014). Cancer patients’ perspectives on multidisciplinary team working: an exploratory focus group study. Urol Nurs.

[12] Borras JM, Albreht T, European Partnership Action Against Cancer consensus group (2014). Policy statement on multidisciplinary cancer care. Eur J Cancer Oxf Engl.

[13] Snyder J, Schultz L, Walbert T (2017). The role of tumor board conferences in neuro-oncology: a nationwide provider survey. J Neurooncol.

[14] Riquet M, Mordant P, Henni M (2013). Should all cases of lung cancer be presented at tumor board conferences?. Thorac Surg Clin.

[15] Kostaras X, Shea-Budgell MA, Malcolm E (2012). Is there a role for clinical practice guidelines in multidisciplinary tumor board meetings? A descriptive study of knowledge transfer between research and practice. J Cancer Educ.

[16] Eusoma (2000). The requirements of a specialist breast unit. Eur J Cancer.

[17] Chan WF, Cheung PSY, Epstein RJ (2006). Multidisciplinary approach to the management of breast cancer in Hong Kong. World J Surg.

[18] Brandão M, Guisseve A, Bata G (2021). Survival impact and cost-effectiveness of a multidisciplinary tumor board for breast cancer in Mozambique, Sub-Saharan Africa. Oncologist.

[19] Henderson F, Lepard J, Seibly J (2021). An online tumor board with international neurosurgical collaboration guides surgical decision-making in Western Kenya. Childs Nerv Syst.

[20] George PE, Fahdil G, Luutu I (2019). Analysis of management decisions and outcomes of a weekly multidisciplinary pediatric tumor board meeting in Uganda. Future Sci OA.

[21] El Saghir NS, Adebamowo CA, Anderson BO (2011). Breast cancer management in low resource countries (LRCs): consensus statement from the breast health global initiative. Breast Edinb Scotl.

[22] Vanderpuye VDNK, Olopade OI, Huo D (2017). Pilot survey of breast cancer management in Sub-Saharan Africa. J Glob Oncol.

[23] Srivastava A, Jalink M, de Moraes FY (2021). Tracking the workforce 2020–2030: making the case for a cancer workforce registry. JCO Glob Oncol.

[24] Team IMB (2012). Revolutionary cancer care in Rwanda. Inshuti Mu Buzima.

[25] Rubagumya F, Greenberg L, Manirakiza A (2017). Increasing global access to cancer care: models of care with non-oncologists as primary providers. Lancet Oncol.

[26] Rubagumya F, Costas-Chavarri A, Manirakiza A (2020). State of cancer control in Rwanda: past, present, and future opportunities. JCO Glob Oncol.

[27] Price AJ, Ndom P, Atenguena E (2012). Cancer care challenges in developing countries. Cancer.

[28] Urquhart R, Kendell C, Sargeant J (2012). How do surgeons decide to refer patients for adjuvant cancer treatment? Protocol for a qualitative study. Implement Sci.

[29] Rubagumya F, Mitera G, Ka S (2020). Choosing wisely Africa: ten low-value or harmful practices that should be avoided in cancer care. JCO Glob Oncol.

[30] Tumor Boards: Optimizing the Structure and Improving Efficiency of Multidisciplinary Management of Patients with Cancer Worldwide. American Society of Clinical Oncology Educational Book..

[31] Grover S, Chiyapo SP, Puri P (2017). Multidisciplinary gynecologic oncology clinic in Botswana: a model for multidisciplinary oncology care in low- and middle-income settings. J Glob Oncol.

[32] Charara RN, Kreidieh FY, Farhat RA (2017). Practice and impact of multidisciplinary tumor boards on patient management: a prospective study. J Glob Oncol.

[33] Soukup T, Lamb BW, Arora S (2018). Successful strategies in implementing a multidisciplinary team working in the care of patients with cancer: an overview and synthesis of the available literature. J Multidiscip Healthc.

[34] American College of Surgeons (2016). Cancer Program Standards: Ensuring Patient-Centered Care.

[35] Chang JH, Vines E, Bertsch H (2001). The impact of a multidisciplinary breast cancer center on recommendations for patient management: the University of Pennsylvania experience. Cancer.

[36] Newman EA, Guest AB, Helvie MA (2006). Changes in surgical management resulting from case review at a breast cancer multidisciplinary tumor board. Cancer.

[37] Petty JK, Vetto JT (2002). Beyond doughnuts: tumor board recommendations influence patient care. J Cancer Educ.

[38] Kehl KL, Landrum MB, Kahn L (2015). Tumor board participation among physicians caring for patients with lung or colorectal cancer. J Oncol Pract.

[39] Stevenson MM, Irwin T, Lowry T (2013). Development of a virtual multidisciplinary lung cancer tumor board in a community setting. J Oncol Pract.

[40] Sarff M, Rogers W, Blanke C (2008). Evaluation of the tumor board as a continuing medical education (CME) activity: is it useful?. J Cancer Educ.

